# Baseline Characteristics of the Paediatric Observation Priority Score in Emergency Departments outside Its Centre of Derivation

**DOI:** 10.1155/2017/9060852

**Published:** 2017-07-24

**Authors:** Damian Roland, Fawaz Arshad, Tim Coats, Ffion Davies

**Affiliations:** ^1^Paediatric Emergency Medicine Leicester Academic (PEMLA) Group, Emergency Department, Infirmary Square, Leicester LE1 5WW, UK; ^2^SAPPHIRE Group, Health Sciences, Leicester University, Leicester, UK; ^3^Emergency Medicine Academic Group, Cardiovascular Sciences, Leicester University, Leicester, UK

## Abstract

**Objectives and Background:**

Scoring systems in Emergency Departments (EDs) are rarely validated. This study aimed to examine the Paediatric Observation Priority Score (POPS), a method of quantifying patient acuity, in EDs in the United Kingdom, and determine baseline performance characteristics.

**Methods:**

POPS was implemented in 4 EDs for children (ages of 0 to 16) with participants grouped into 3 categories: discharged from ED, discharged but with return within 7 days, and admitted for less or more than 24 hours.

**Results:**

3323 participants with POPS scores ranging from 0 to 11 (mean = 2.33) were included. The proportion of each POPS score varied between sites with approximately 10–20% being POPS 0 and 12–25% POPS greater than 4. Odds ratio of readmission with POPS 5–9 against 0–4 was 2.05 (CI 1.20 to 3.52). POPS 0–4 showed no significant difference (*p* = 0.93) in relation to admission/discharge rates between sites with a significant difference found (*p* < 0.01) for POPS > 5.

**Conclusion:**

It is feasible to implement POPS into EDs with similar performance characteristics to the original site of development. There is now evidence to support a wider health service evaluation to refine and improve the performance of POPS.

## 1. Background

In developed countries serious childhood illness is uncommon but increasing numbers of families seek urgent face-to-face medical assessment [1, 2] for minor illness, and there continue to be missed opportunities in staff identifying seriously ill children [3]. Risk-averse strategies of referring all children of “potential concern” for specialist paediatric assessment overload an already stretched out-of-hours system and lead to unnecessary hospital admissions. Rational and efficient healthcare requires there to be an objective system of measuring how unwell a child is. Although “scoring tools” exist most are ward-based “early warning scores (EWS)” which identify children needing high-level critical care. They are not validated for use early in illness [4], or through the range of childhood acute illness (mild to severe). Attempts to implement ward-based scoring systems have resulted in disappointing results [5, 6].

The Paediatric Observation Priority Score (POPS) is a method of identifying the range of severity of childhood illness, to support staff in taking the decision to redirect the child to primary care or discharge to self-care and to help them in expediting senior/specialist assistance for deteriorating children [7]. POPS is not intended to determine a course of action but to support clinical decisions, especially for staff not familiar with dealing with child.

**Figure 1:**
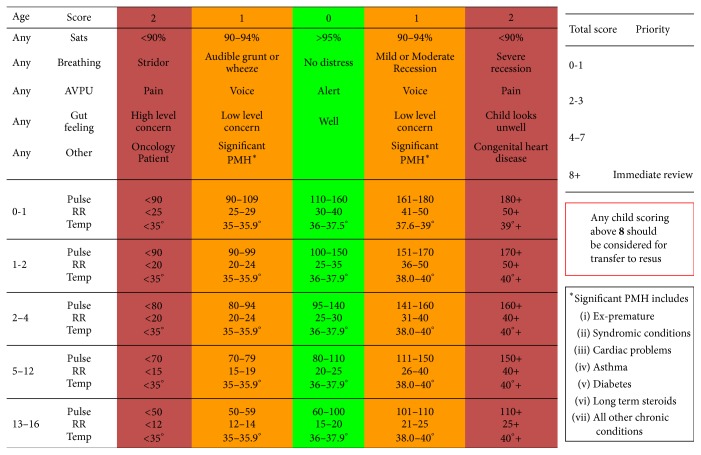
Candidate items evaluated for POPS. Paediatric Observation Priority Score (POPS) Chart. This chart is not a substitute for good clinical judgement and any concerns about the condition of a child should be brought to the attention of a senior nurse or doctor. POPS is copyrighted (creative commons attribution noncommercial share-alike 4.0), Dr. Damian Roland and Dr. Ffion Davies 2010. This is version 1.3, August 2016.

POPS uses a combination of physiological, behavioural, and known-risk parameters to generate a score of either 0, 1, or 2 (Figure 1), giving a total score (0–16). POPS has undergone an initial single centre validation in a cohort of 942 patients [8], a larger group than any previous Emergency Department (ED) study [9]. Further local data on over 20,000 patients has demonstrated a relationship between length of admission and increasing POPS, with an increase when POPS > 4, and good safety profile [10].

Scoring systems often perform less well outside the organisation that developed the score. This study aimed to validate how the POPS score performed in other EDs in the United Kingdom (UK).

## 2. Methods

The objectives of the study were to describe the distribution of scores in EDs outside our institution, confirm a threshold indicator of POPS > 4 for hospital admission risk (which was the threshold in local data), and examine discharge and representation rates following uptake of POPS. POPS was implemented in 4 EDs. The characteristics of each of the EDs is shown in Table 1. The implementation and education of local staff about POPS was left to the discretion of the local team.

All children (0–16 years) presenting to these departments were included on a convenience basis from February 2013 to June 2013. The department's normal patient assessment/triage procedure was followed as per local protocol with the POPS observations being taken at the first available opportunity. National Standards in UK require an initial assessment of patients to occur within 15 minutes of arrival which all departments worked towards. A report form was used for prospective collection of each child's POPS score (Figure 1), age, and unique study identifier. The local research team later recorded whether or not the patient was admitted, length of admission, and representation in the next 7 days.

For the analysis patients were grouped into those that (a) were discharged from ED (including outpatient referrals) and who did not return within 7 days, (b) were admitted for less than 24 hours or represented within 7 days after discharge, and (c) were admitted to hospital for more than 24 hours. For validation of the admission threshold patients were separated into two groups based on an odds of admission calculated for the groups above and below a POPS of 4.

Ethical approval was granted via the East Midlands REC committee 2 and need for consent waived. Descriptive and statistical data were examined using SPSS v22.0.

## 3. Results

**Figure 2:**
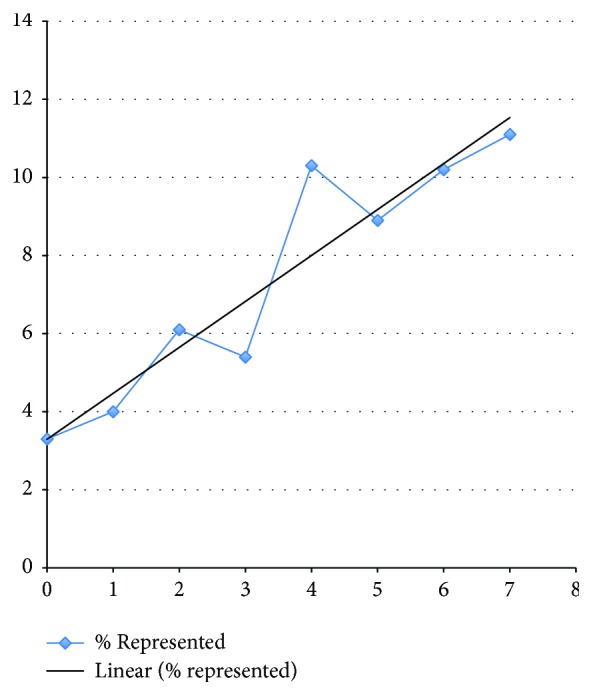
Relationship between readmission rate and POPS score on initial presentation.

**Figure 3:**
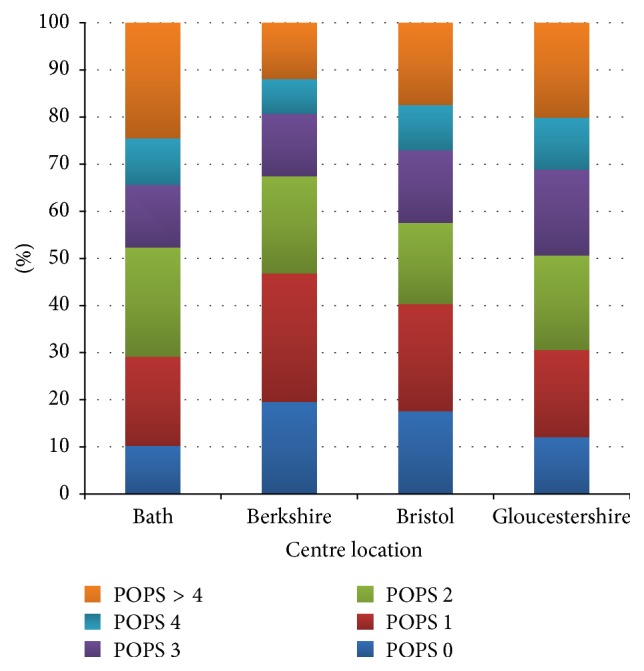
Proportion of POPS at each site.

**Figure 4:**
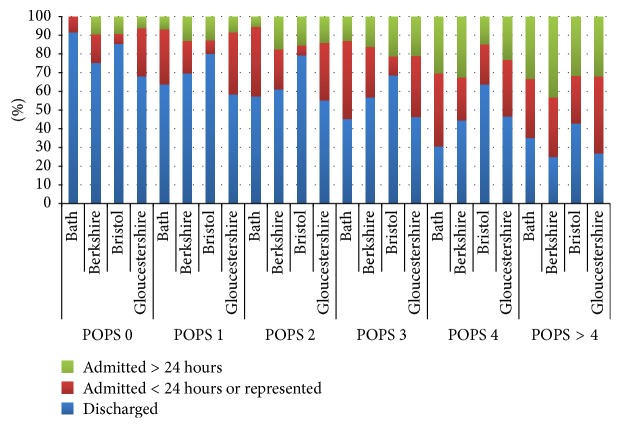
Disposition of patients presenting to 4 separate EDs, according to POPS.

Of 3338 patients submitted 15 were excluded (7 self-discharged and 8 unclear outcomes). The remaining patients are described in Table 1. Overall discharge rate was 61.7% across all sites with a range of 49.1% to 72.1% at individual sites. 111 (5.4%) of patients were represented (Table 2) with increasing representations rates as POPS increases, that is, a trend to being linear (Figure 2). POPS scores ranged from 0 to 11 with a median score 2 and interquartile range of 2. The POPS scores in the 4 sites are shown in Figure 3. The proportion of each POPS score varied between sites with approximately 10–20% being POPS 0 and 12–25% POPS greater than 4. Admission rates (both for less than and more than 24 hours) increased as POPS increased in all 4 sites (Figure 4). In relation to admission and discharge the receiver operating characteristic (ROC) curve area across all sites for admission was 0.66 (CI 0.64 to 0.69).

In relation to threshold analysis across all centres, with POPS dichotomised into 0–4 and 5–11, odds ratio of admission was 5.13 (CI 4.06 to 6.49). Controlling for location there was no significant difference in the odds ratios for POPS 0–4 (*p* = 0.93). However, there was a significant difference between admission/discharge for POPS > 5 (*p* < 0.01) (Table 3). Odds ratio of readmission with POPS 5–9 against 0–4 was 2.05 (CI 1.20 to 3.52).

## 4. Discussion

This data demonstrates it is feasible to introduce POPS into EDs and maintain similar performance characteristics between sites. Across all sites POPS performed moderately well at predicting admission and safe discharge with similar characteristics to the site of derivation. Despite no specific education in the use of POPS, its performance was similar between sites with some expected variation due to different populations and size of units. Its performance, despite the absence of a well-defined, implementation strategy, would suggest it is a system that is relatively easy to implement successfully.

Unlike inpatient Early Warning Scores designed to detect deterioration, POPS has demonstrated an ability to aid detection of patients requiring hospitalisation in the ED safely, while also supporting discharge decision making for children with minor illness. The strong relationship between return rates and initial POPS allows a clinician or individual unit to define their own management thresholds, depending on their attitude to risk. It may be that children with intermediate POPS scores (3–6) would benefit from a mandated senior review prior to discharge, and identifying this middle group would assist each site in fine-tuning their discharge/admission threshold. At POPS above 4 in all the sites 30% of children were admitted for over 24 hours' rate. An admission for over 24 hours infers that ongoing management was deemed necessary by the paediatric team and admission was unlikely to be related to any service or capacity pressures. While an individual score should never on its own determine the decision of a child these results support the use of a cut-off of around 4 as a decision making aid for admission and discharge. Senior clinicians could obviously make their own adjustments depending on local factors.

There are many potential benefits to wide scale implementation of this system aside from the rapid detection of critically unwell children. Potential cost savings include fewer referrals for inpatient treatment (current risk-averse practice results in overreferral), fewer serious adverse events (missed serious illness), and reducing numbers or costs of litigation claims. The combination of POPS with inpatient EWS is being investigated and will further enhance serious illness detection in the future (personal communication).

Although data was collected prospectively the time periods were not uniform between sites and there was no measure to determine standardisation of assessment between staff in a given Emergency Department. The ROC score remains low but comparable with other Paediatric Early Warning Scores used in children's Emergency Departments [5] and an external group have utilised a modified version of POPS and demonstrated its predictive value and safety profile [11]. It is not the intention for the POPS to be decision making arbiter, more support of the assessment of a child, especially for junior medical and nursing staff.

## 5. Conclusion

We have demonstrated it is feasible to implement POPS into several EDs. This data can now be used to inform a formal prospective external validation study and assist in ongoing rollout to interested sites.

## Figures and Tables

**Table 1:** Patient disposition at individual hospital sites during study period.

Location (annual attendance of children to nearest 1000)		Successful ED discharge		Admitted < 24 hours or represented		Admitted > 24 hours		Total
Hospital location	Bath (12000)	122	52.4%	75	32.2%	36	15.5%	**233**
	Berkshire (26000)	1034	60.2%	364	21.2%	321	18.7%	**1719**
	Bristol (35000)	702	72.1%	110	11.3%	170	17.5%	**973**
	Gloucestershire (15000)	191	49.1%	128	32.9%	70	18.0%	**389**

Combined		2049	61.7%	677	20.4%	597	18.0%	**3323**

**Table 2:** Table of discharges and representations.

POPS	Successful discharge	Discharged but represented	Percentage represented
0	457	15	3.3
1	576	23	4.0
2	425	26	6.1
3	280	15	5.4
4	145	15	10.3
5	90	8	8.9
6	49	5	10.2
7	18	2	11.1
8	7	2	28.6
**9**	**2**	**0**	**0.0**

Total	2049	111	5.4

**Table 3:** Odds ratio for admission at each site for POPS 5–11 against 0–4.

Location	Odds ratio for admission
Bath	5.70 (CI 2.53 to 12.83)
Berkshire	7.28 (CI 5.01 to 10.57)
Bristol	3.95 (CI 2.64 to 5.91)
Gloucestershire	4.50 (CI 2.31 to 8.76)

Combined	5.13 (CI 4.06 to 6.49)
